# MicroRNA-21: An Emerging Player in Bone Diseases

**DOI:** 10.3389/fphar.2021.722804

**Published:** 2021-09-07

**Authors:** Chen Chen, Ya-Mei Liu, Bin-Lan Fu, Liang-Liang Xu, Bin Wang

**Affiliations:** ^1^ School of Basic Medical Science, Guangzhou University of Chinese Medicine, Guangzhou, China; ^2^ Laboratory of Orthopaedics and Traumatology, Lingnan Medical Research Center, Guangzhou University of Chinese Medicine, Guangzhou, China; ^3^ Key Laboratory of Orthopaedics and Traumatology, The First Affiliated Hospital of Guangzhou University of Chinese Medicine, Guangzhou University of Chinese Medicine, Guangzhou, China; ^4^ Department of Traumatology, the Third Affiliated Hospital of Guangzhou University of Chinese Medicine, Guangzhou, China

**Keywords:** miRNAs, miR-21, osteoblasts, osteoclasts, bone diseases

## Abstract

MicroRNAs (MiRNAs) are small endogenous non-coding RNAs that bind to the 3′-untranslated region of target genes and promote their degradation or inhibit translation, thereby regulating gene expression. MiRNAs are ubiquitous in biology and are involved in many biological processes, playing an important role in a variety of physiological and pathological processes. MiRNA-21 (miR-21) is one of them. In recent years, miR-21 has received a lot of attention from researchers as an emerging player in orthopedic diseases. MiR-21 is closely associated with the occurrence, development, treatment, and prevention of orthopedic diseases through a variety of mechanisms. This review summarizes its effects on osteoblasts, osteoclasts and their relationship with osteoporosis, fracture, osteoarthritis (OA), osteonecrosis, providing a new way of thinking for the diagnosis, treatment and prevention of these bone diseases.

## Introduction

**FIGURE 1:**
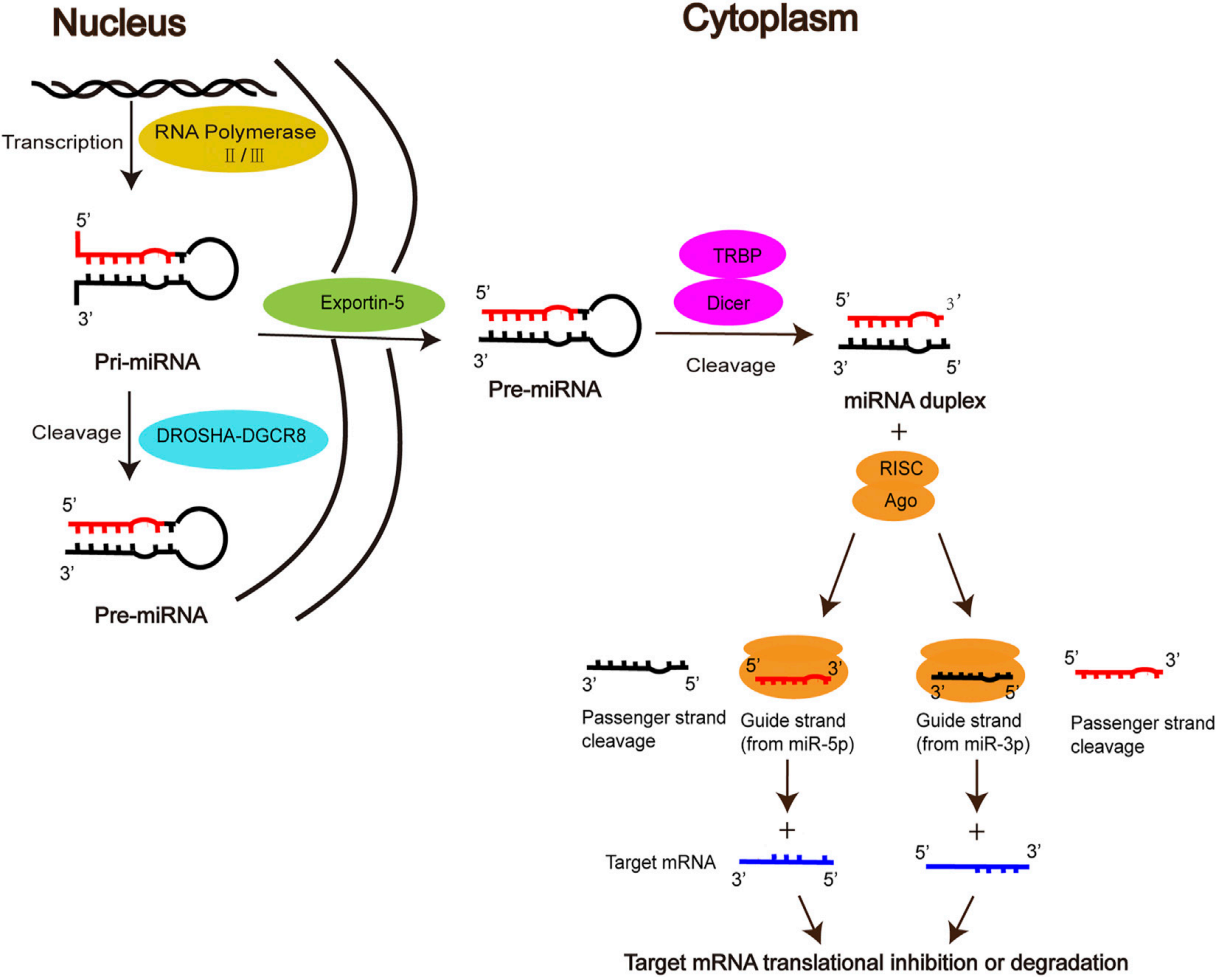
Biogenesis and function of miRNAs.

MiRNAs, first discovered by Lee et al. in *elegans*, are a class of endogenous non-coding small RNAs that play an important regulatory role in gene expression at the post-transcriptional level (Lee et al., 1993; Zhang et al., 2012). The biogenesis of mature miRNA undergoes the following stages. Firstly, primary miRNA (pri-miRNA) is produced in the nucleus by RNA polymerase II/III, then cleaved into precursor miRNA (Pre-miRNA) by the DROSHA-DGCR8 complex, which is exported via the nuclear membrane protein Exportin5 to the cytoplasm. In the cytoplasm, Pre-miRNA is further processed by the RNase III enzyme Dicer and the double-stranded RNA-binding domain proteins TRBP into miRNA duplex, one strand of which is referred to as the miR-5p strand and the other as the miR-3p strand. The duplex is incorporated into the RNA-induced silencing complex (RISC), in which Argonaute (AGO) directs the mature strand (guide strand) to bind to the complementary site in the 3′ untranslated region of the target mRNA, leading to degradation of the target mRNA or inhibition of translation of the target mRNA, thereby regulating gene expression (Sayed and Abdellatif, 2011; Takada and Asahara, 2012; Lo et al., 2013; Báez-Vega et al., 2016; Michlewski and Caceres, 2019; Gareev et al., 2020). The other strand (passenger strand), on the other hand, is degraded due to its low steady-state level. However, there is growing evidence that passenger strands can be accumulated to substantial levels and can also play biological roles as microRNAs (Guo and Lu, 2010; Lo et al., 2013; Doberstein et al., 2014) (Figure 1).

MiRNAs are ubiquitous in all organisms, and it is estimated that miRNAs regulate approximately more than 60% of protein-coding genes (Smirnova et al., 2014), indicating that miRNAs play a vital role in a variety of physiological and pathological processes. Currently, there is increasing evidence that miRNAs are involved in skeletal development and play an important role in regulating bone homeostasis (Gamez et al., 2014; Zhao et al., 2014). Also miRNAs are closely related to a variety of orthopedic diseases, which includes participation in the occurrence and development of bone diseases, and have an impact on the diagnosis, treatment and prognosis of bone diseases (Gordon et al., 2014; Gennari et al., 2017; Yang and Fang, 2017; Feichtinger et al., 2018; van Meurs et al., 2019). MiR-21 is one of the miRNAs that has received a lot of attention as an emerging player in bone diseases. Therefore, this article will review the relationship between miR-21 and osteoblasts, osteoclasts, osteoporosis, fractures, OA and osteonecrosis.

## MiR-21 Regulates Osteogenic Differentiation

MiR-21 has a regulatory role in osteogenic differentiation. As we all know, mesenchymal stem cells (MSCs) are a kind of multipotent stem cell with the potential of multi-directional differentiation (Lin et al., 2019). It is the precursor cell of osteoblasts and osteocytes in the process of bone formation (Hou et al., 2021). A variety of mechanisms are involved in regulating the differentiation of MSCs into osteoblasts and osteocytes by miR-21. MiR-21 has been reported to activate the ERK-MAPK signaling pathway, and activation of the ERK-MAPK signaling pathway can promote osteogenesis in MSCs. For example, Yang et al. found that miR-21 promoted osteogenic differentiation of MSCs by suppressing the expression of its target gene SPRY1, a process that indirectly activates the ERK-MAPK signaling pathway (Yang et al., 2013). MiR-21 maintains the activation of ERK-MAPK signaling by decreasing the level of SPRY2, thereby increasing the expression of osteogenic differentiation-related transcription factors (Mei et al., 2013). Valproic acid, a flavonoid, activates the ERK/MAPK signaling pathway by upregulating the expression of mir-21 in mouse MSCs, thereby promoting the differentiation of mouse MSCs into osteoblasts (Akshaya et al., 2021). Furthermore, in fetal amniotic fluid-derived MSCs, induction of miR-21 expression accelerates bone formation, a process associated with miR-21 suppressing the expression of the transcription factor SOX2 and regulating the differentiation properties of amniotic fluid-derived MSCs (Trohatou et al., 2014). In human umbilical cord mesenchymal stem cells (HUMSCs), miR-21 promotes osteogenic differentiation by inhibiting PTEN and activating the PI3K-AKT-GSK3β pathway (Meng et al., 2015). MiR-21 can also promote osteogenic differentiation of bone marrow mesenchymal stem cells (BMMSCs) by targeting the SMAD7-SMAD1/5/8-RUNX2 pathway (Li et al., 2017).

In addition, miR-21 positively regulates the osteogenic differentiation of MC3T3-E1 cells, murine multilineage cells (MMCs) and periodontal ligament cells (PDLCs). In MC3T3-E1 cells, icariin attenuated the inhibitory effect of titanium particles on osteoblast differentiation and matrix mineralization by upregulating miR-21-5p (A mature sequence from the 5′ end of the miR-21 stem-loop precursor for the miR-21 guide strand) expression, revealing the promotional role of miR-21-5p in osteoblast differentiation and mineralization (Lian et al., 2018). Oka et al. further demonstrated that miR-21 positively regulated osteogenic differentiation and mineralization by promoting the expression of key osteogenic factors ALP, RUNX2, OPN and OSX in MC3T3-E1 cells, and this result was verified in miR-21 knockout mice (Oka et al., 2021). Similarly, in MC3T3-E1 cells, miR-21 promotes osteogenic differentiation by inhibiting the translation of SMAD7 protein (Li et al., 2015). And in MMCs cells, SONG et al. further showed that by upregulating miR-21 expression, the level of SMAD7 could be reduced to maintain the activation of BMP9/SMAD signaling, thus promoting osteogenic differentiation (Song et al., 2015). In 2012, Li et al. initially found that miR-21 regulates the expression of PLAP-1, and they are inversely correlated, while PLAP-1 plays a negative role in osteogenic differentiation in maintaining the homeostasis of the periodontium. This finding suggests that miR-21 may be involved in the osteogenic differentiation of PDLCs (Li et al., 2012). Later, miR-21 was further demonstrated to promote stretch-induced osteogenic differentiation in human periodontal ligament stem cells (HPDLSCs), and this effect may be achieved by miR-21 inhibiting the expression of its target gene ACVR2B (Wei et al., 2015). Also in HPDLSCs, tumor necrosis factor-α (TNF-α) inhibition of miR-21 expression impaired osteogenic differentiation. The elevated SPRY1 level caused by inhibition of miR-21 may be one of the reasons for the impaired osteogenic differentiation in HPDLSCs (Yang et al., 2017), which is consistent with the previous finding of Yang et al. in MSCs (Yang et al., 2013).

**FIGURE 2:**
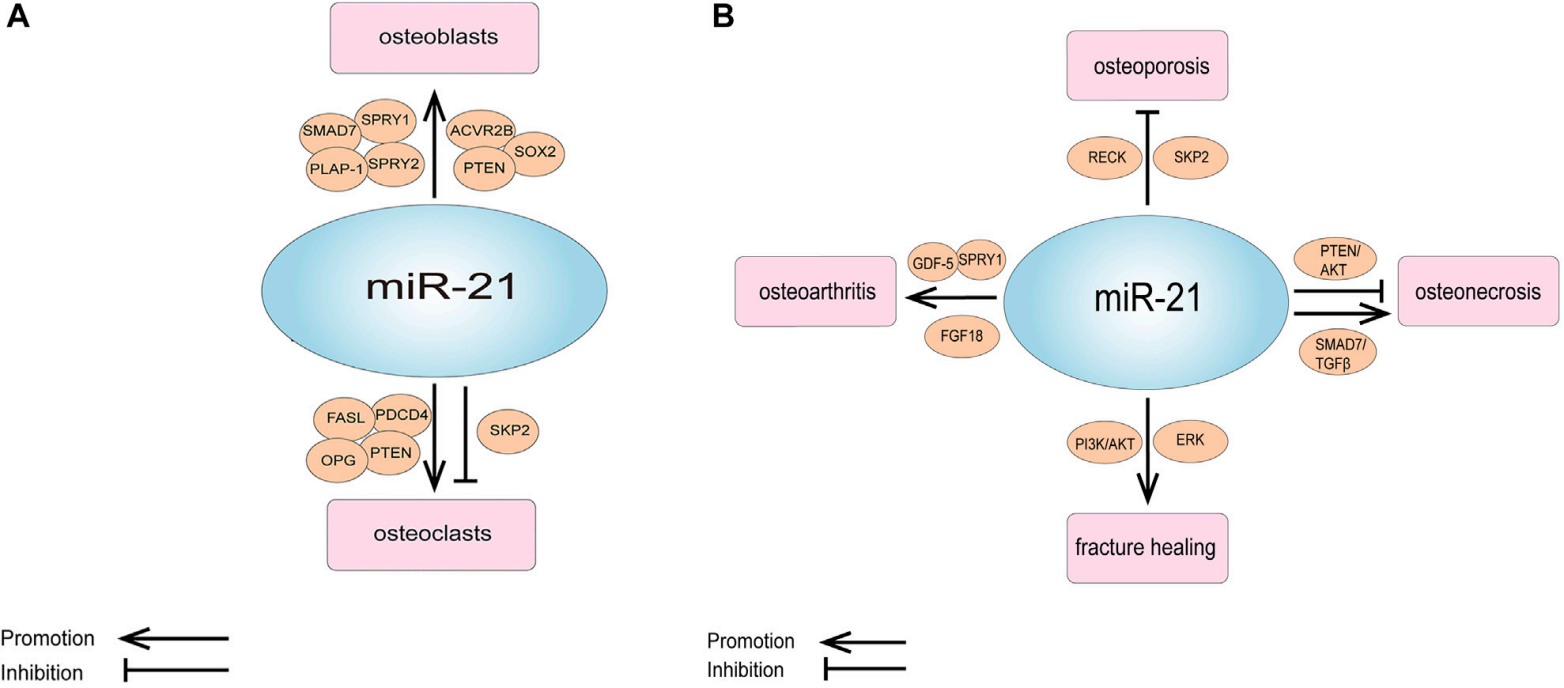
**(A)** Schematic representation of the role of miR-21 and its targets in osteogenesis and osteoclastic. **(B)** Schematic representation of the role of miR-21 and its targets in orthopedic diseases.

However, some of the studies were not consistent with these results. Sheng et al. detected that downregulation of miR-21 in a rabbit tibial fracture model promoted osteoblast proliferation by positively regulating the expression of growth factors downstream of the ERK signaling pathway (Sheng et al., 2019). MiR-21-5p was raised in glucocorticoid-induced rat BMMSCs, and the overexpression of miR-21-5p significantly suppressed osteogenic differentiation and proliferation of BMMSCs and promoted apoptosis (Hao et al., 2021) (Table 1; Figure 2A).

**TABLE 1:** Targets of miR-21 for osteogenic differentiation.

Experimental model	Gene target	Effects	References
MSCs	SPRY1	promotes MSCs osteogenesis through the miR-21/SPRY1 functional axis	Yang et al. (2013)
MSCs	SPRY2	inhibits the expression of SPRY2, activates ERK-MAPK signal pathway, and increases the level of transcription factors related to osteogenic differentiation	Mei et al. (2013)
MSCs	SOX2	inhibits the expression of SOX2 and accelerates osteogenesis	Trohatou et al. (2014)
MSCs	PTEN	activates the PI3K-AKT-GSK3β pathway and promotes the entry of β-catenin into the nucleus, thus promoting osteogenic differentiation	Meng et al. (2015)
MSCs	SMAD7	promotes osteogenic differentiation through SMAD7-SMAD1/5/8-RUNX2 pathway	Li et al. (2017)
MC3T3-E1	SMAD7	promotes osteogenic differentiation and mineralization by inhibiting the expression of SMAD7	Li et al. (2015)
MMCs	SMAD7	promotes osteogenic differentiation by reducing the level of SMAD7 to maintain the activation of BMP9/SMAD signal	Song et al. (2015)
PDLCs	PLAP-1	promotes the osteogenic differentiation of PDLCs by regulating the expression of PLAP-1	Li et al. (2012)
HPDLSCs	ACVR2B	promotes stretch-induced osteogenic differentiation by inhibiting the expression of ACVR2B	Wei et al. (2015)

## MiR-21 Regulates Osteoclastic Differentiation

The regulatory effects of miR-21 on osteoclasts are complex and involve multiple mechanisms. First, the study by Sugatani et al. showed that miR-21 expression was upregulated during RANKL-induced osteoclastogenesis, while they proposed a new molecular mechanism regarding osteoclastogenesis, namely the C-Fos/miR-21/PDCD4 positive feedback loop. C-Fos upregulated miR-21 expression and downregulated PDCD4 protein expression, and attenuated PDCD4 eliminated the inhibitory effect of C-Fos, which in turn promoted osteoclastogenesis (Sugatani et al., 2011). This positive feedback loop was also validated in the mouse model of particle-induced osteolysis (Zhou et al., 2012). Later, using miR-21 knockout mice models, miR-21 was shown to promote bone resorption *in vivo* by directly regulating osteoclast function through targeting PDCD4 (Hu et al., 2017). Lung adenocarcinoma cell-derived exosomal miR-21 also promotes osteoclast formation by targeting PDCD4 (Xu et al., 2018). In periodontal accelerated osteogenesis orthodontics (PAOO) facilitated orthodontic tooth movement (TM), the positive feedback loop of C-Fos/miR-21/PDCD4 leads to an increase in osteoclast production, thus increasing movement of orthodontic teeth (Zhang Y. et al., 2020). Second, Wang et al. confirmed by bioinformatics and dual-luciferase reporter assays that PTEN is a target gene of miR-21, which promotes osteoclast formation and bone resorption by targeting Pten to activate the PI3K/AKT signaling pathway (Wang et al., 2020). Circulating miR-21 of lung cancer cell origin also targets PTEN and promotes osteoclastogenesis (Zhao et al., 2020). However, an inhibitory effect of miR-21 on osteoclast differentiation has also been reported, for instance, Huang et al. investigated that miR-21-5p was significantly decreased during osteoclast differentiation and that miR-21-5p inhibited osteoclast differentiation by acting on its target gene SKP2 (Huang et al., 2021). In juvenile idiopathic arthritis, miR-21 could inhibit the production of osteoclasts induced from rheumatoid arthritis fibroblast-like synovial cells by M-CSF (Li and Zeng, 2020).

MiR-21 also affects osteoclasts by influencing the RANKL/OPG ratio. It was shown that OPG is a potential target of miR-21. MiR-21 expression was significantly enhanced in bone marrow stromal cells (BMSCs) adherent to multiple myeloma cells, while OPG expression was significantly reduced. Inhibition of miR-21 expression restored the RANKL/OPG balance and significantly impaired the resorptive activity of mature osteoclasts (Pitari et al., 2015). In the miR-21 knockout mice orthodontic TM model, miR-21 was revealed to enhance osteoclast differentiation by inducing RANKL secretion from activated T cells, which in turn regulated the RANKL/OPG balance and partially relieved the decreased orthodontic TM distance in miR-21 knockout mice (Wu et al., 2020). A study by Li et al. yielded different findings. They demonstrated that in the presence of mechanically induced maxillary expansion, by comparing miR-21 knockout and wild-type mice, it was found that miR-21 deficiency induced more osteoclasts by downregulating OPG expression and upregulating RANKL expression. And after intraperitoneal injection of agomir-21 to miR-21 knockout mice, the OPG/RANKL ratio was rescued (Li et al., 2020). Clinical studies have discovered that serum miR-21 is positively correlated with RANKL levels and negatively correlated with OPG levels in postmenopausal hypoestrogenic women with osteoporosis (PMOP). Moreover, MiR-21 overexpression leads to an increase in RANKL/OPG ratio, and a higher RANKL/OPG ratio increases the number of osteoclasts and bone resorption activity (Suarjana et al., 2019).

MiR-21 is also involved in osteoclast apoptosis. It has been reported that estrogen causes osteoclast apoptosis by down-regulating miR-21 biogenesis and increasing the post-transcriptional levels of the FasL protein, the target of miR-21 (Sugatani and Hruska, 2013). MiR-21 was also found to interact directly with lncRNA GAS5 to promote apoptosis in osteoclasts (Cong et al., 2020) (Table 2; Figure 2A).

**TABLE 2:** Targets of miR-21 that affect osteoclast differentiation.

Experimental model	Gene target	Effects	References
Murine bone marrow monocytes and mice animal model	PDCD4	promotes osteoclastogenesis through a positive feedback loop of C-Fos/miR-21/PDCD4	Sugatani et al. (2011)
Mice animal model	PDCD4	MiR-21 deficiency inhibits bone resorption and osteoclast function through miR-21 targeting PDCD4	Hu et al. (2017)
Lung adenocarcinoma cell line and Murine bone marrow monocytes	PDCD4	Lung adenocarcinoma cell-derived exosome miR-21 promotes osteoclastogenesis by targeting PDCD4	Xu et al. (2018)
Rat animal model	PDCD4	MiR-21 negatively regulates the target gene PDCD4, leading to increased expression of C-Fos and increased RANKL-mediated osteoclastogenesis	Zhang et al. (2020b)
Macrophage cell line RAW264.7	PTEN	MiR-21 promotes osteoclast formation and bone resorption by targeting Pten to activate the PI3K/AKT signaling pathway	Wang et al. (2020)
Human small cell lung cancer (SCLC) cells, macrophage cell line RAW264.7 and Human primary monocytes	PTEN	Circulating miR-21 of lung cancer cell origin promotes osteoclastogenesis via PTEN.	Zhao et al. (2020)
Bone marrow macrophages, macrophage cell line RAW264.7 and mice animal model	SKP2	MiR-21-5p inhibited osteoclast differentiation by acting on its target gene SKP2	Huang et al. (2021)
Bone marrow stromal cells, multiple myeloma cells and peripheral blood mononuclear cells	OPG	Inhibition of miR-21 expression restored the balance of RANKL/OPG and significantly weakened the absorptive activity of mature osteoclasts	Pitari et al. (2015)
Murine bone marrow monocytes and mice animal model	FasL	Estrogen promotes osteoclast apoptosis by down-regulating miR-21 biogenesis while post-transcriptionally increasing FasL production	Sugatani and Hruska, (2013)

## Potential Role of miR-21 in Diagnosis and Treatment of Osteoporosis

Osteoporosis is a bone disease with a high prevalence, which is common in the elderly, especially in postmenopausal women. Osteoporosis is characterized by decreased bone mass and destruction of bone tissue microstructure, resulting in increased bone brittleness and fracture risk. It has brought a serious economic burden to human society (Wu et al., 2021). The clinical diagnosis of osteoporosis is mainly based on bone mineral density (BMD). Low BMD often increases the risk of osteoporosis. The imbalance between osteoblast-induced bone formation and osteoclast-induced bone resorption plays an important role in the pathogenesis of osteoporosis (Khosla et al., 2011; Lu et al., 2021). MiR-21 has effects on both osteoblasts and osteoclasts, so miR-21 may also play an important role in osteoporosis.

In laboratory studies, Zhao et al. showed that miR-21 regulates osteoporosis by targeting RECK. They established a cell model of osteoporosis by adding TNF-α to the medium of MSCs and found that miR-21 mimics as well as RECK siRNA attenuated the effects of TNF-α on apoptosis, proliferation and differentiation of MSCs, and increased the expression of MT1-MMP. A luciferase reporter gene assay showed that RECK was a direct target of miR-21. They further used ovariectomy (OVX) mouse model of osteoporosis, and discovered that the expression of miR-21 decreased while RECK increased in the OVX mice; when treatment with lentiviral RECK shRNA, the osteoporosis of OVX mice could be inhibited (Zhao et al., 2015). In addition, miR-21 can also regulate osteoporosis by affecting osteoclastogenesis, as mentioned earlier in the study by Huang et al. who noticed that miR-21-5p targeting SKP2 inhibited osteoclast differentiation. They also revealed that miR-21-5p treatment inhibited bone resorption and maintained bone cortex and trabecular structures (Huang et al., 2021). All of these results suggest that miR-21 is a new target for the treatment of osteoporosis (Figure 2B; Table 3).

**TABLE 3:** Targets of miR-21 in bone diseases.

Types of bone diseases	Experimental model	Gene target	References
Osteoporosis	MSCs and mice animal model	RECK	Zhao et al. (2015)
Osteoporosis	Bone marrow macrophages, macrophage cell line RAW264.7 and mice animal model	SKP2	Huang et al. (2021)
Fracture	Rat animal model	PI3K/AKT signaling pathway	Liu et al. (2019)
Fracture	Rabbit animal model	ERK signaling pathway	Sheng et al. (2019)
OA	Chondrocyte cell line	GDF-5	Zhang et al. (2014)
OA	Human chondrocytes and mice animal model	FGF18	Wang et al. (2019)
TMJOA	Mouse condylar chondrocytes and mice animal model	SPRY1	Ma et al. (2020)
TMJOA	Mandibular condylar chondrocytes and mice animal model	GDF-5	Zhang et al. (2020a)
SIONFH	BMSCs and rat animal model	Smad7/TGFβ signaling pathway	Hao et al. (2021)
GIONFH	HWJ-MSCs, murine osteocyte-like MLO-Y4 cells and rat animal model	PTEN/AKT signal pathway	Kuang et al. (2019)

In clinical studies, miR-21 is differentially expressed in patients with osteoporosis and may serve not only as a new biomarker for the diagnosis of osteoporosis, but also as a potential target for therapeutic inhibition. Li et al. measured the level of miR-21 in plasma of 120 Chinese postmenopausal women and divided them into normal group, osteopenia group and osteoporosis group. MiR-21 expression was downregulated in plasma of patients with osteoporosis and osteopenia compared with the normal group, and plasma miR-21 levels were positively correlated with BMD (Li et al., 2014). Yavropoulou et al. examined the serum miR-21 level in 100 postmenopausal women and found that the expression of miR-21-5p in serum of postmenopausal women with low bone mass, at least one case of moderate vertebral fracture and low bone mass without fracture was significantly lower than that of the control group with normal BMD and no history of fracture (Yavropoulou et al., 2017). Zhao et al. collected bone tissue and serum from 48 osteoporotic patients and 48 normal subjects, concluded that miR-21 was expressed at low levels in bone tissue and serum in osteoporotic patients (Zhao et al., 2019).

However, the results of some clinical studies are not consistent with the above results. Seeliger et al. and Kelch et al. showed that miR-21-5p was significantly up-regulated in serum and bone tissue of osteoporotic patients, and Kelch et al. also studied miR-21-5p in bone tissue was negatively correlated with BMD (Seeliger et al., 2014; Kelch et al., 2017). Perksanusak et al. analyzed the expression of miR-21 in the plasma of postmenopausal women in Thailand and found that miR-21 expression was significantly higher in the low BMD group (osteopenia and osteoporosis) compared to the normal BMD group, and miR-21-5p was mildly negatively correlated with BMD (Perksanusak et al., 2018). By comparing 60 postmenopausal women with hypoestrogenism with osteoporosis and 60 postmenopausal women with hypoestrogenism without osteoporosis, Suarjana et al. revealed that the expression of serum miR-21 was higher in postmenopausal hypoestrogenism with osteoporosis than in non-osteoporotic patients and was negatively correlated with spinal BMD (Suarjana et al., 2019) (Table 4).

**TABLE 4:** Relevance of miR-21 in bone diseases.

Types of bone diseases	Organs or tissue	Status of miR-21	References
Osteoporosis	Patient sample	Downregulated	Li et al. (2014)
Osteoporosis	Patient sample	Downregulated	Yavropoulou et al. (2017)
Osteoporosis	Patient sample	Downregulated	Zhao et al. (2019)
Osteoporosis, Fracture	Patient sample	Upregulated	Seeliger et al. (2014)
Osteoporosis, Fracture	Patient sample	Upregulated	Kelch et al. (2017)
Osteoporosis	Patient sample	Upregulated	Perksanusak et al. (2018)
Osteoporosis	Patient sample	Upregulated	Suarjana et al. (2019)
Fracture	Patient sample	Upregulated	Panach et al. (2015)
Fracture	Patient sample	Upregulated	Zarecki et al. (2020)
Fracture	Patient sample	Downregulated	Yavropoulou et al. (2017)
OA	Patient sample	Downregulated	Song et al. (2014)
OA	Patient sample	Downregulated	Zhu et al. (2019)
OA	Patient sample	Upregulated	Zhang et al. (2014)
OA	Patient sample	Upregulated	Wang et al. (2019)
OA	Rat animal model	Upregulated	Hoshikawa et al. (2020)
Osteonecrosis	Mice animal model	Upregulated	Wang et al. (2015)
Osteonecrosis	Patient sample, Rat animal model	Upregulated	Yang et al. (2018)
Osteonecrosis	Patient sample	Upregulated	Musolino et al. (2018)
Osteonecrosis	Rat animal model	Upregulated	Hao et al. (2021)

The above findings reveal that the role of miR-21 in regulating osteogenic differentiation and osteoclast formation remains controversial, due to the different regulatory mechanisms. Apart from that, it may also be related to the number of patients involved in each study, different research groups, etc. First of all, the number of patients involved in the above study is relatively small, so it is necessary to further clarify the diagnostic value of miR-21 in osteoporosis in larger samples. Secondly, the subjects selected in the above studies are not the same. For example, fracture patients were included in the studies of Seeliger et al. and Kelch et al. (Seeliger et al., 2014; Kelch et al., 2017), but not in the studies of Li et al. and Zhao et al. (Li et al., 2014; Zhao et al., 2019). Fracture is a complex biological process that leads to an imbalance in the endoskeletal dynamics of the body and also leads to significant changes in the signaling pathways of the molecules involved in the bone reconstruction process, thus having a significant impact on the expression level of miR-21. Also, different inclusion and exclusion criteria for study subjects in each study as well as different methods of measuring outcomes during the study can have an impact on miR-21 expression. Suarjana et al. detected the level of estrogen in the subjects. However, estrogen inhibits miR-21 expression and induces apoptosis in osteoclasts (Sugatani and Hruska, 2013). In summary, due to the inconsistent results of miR-21, more studies are needed to confirm the clinical application of miR-21 to determine whether it can be used as an indicator to assess the occurrence of osteoporosis and as a potential target for the treatment of osteoporosis.

## The Regulatory Role of miR-21 in Fracture

Fracture is a common clinical disease, which refers to the loss of bone integrity, pain, swelling and dysfunction at the fracture site. Although bone has the ability to reconstruct and repair itself, there are still about 5–10% of fractures result in delayed unions or non-unions. The disorder of fracture healing will cause not only personal losses, but also economic losses (Jayankura et al., 2021). Therefore, it is necessary to deeply study the mechanism of promoting fracture healing in order to lighten the burden for human society.

As we all know, Fracture healing is a complex biological process, which includes the inflammation stage, callus stage and bone remodeling stage (Hadjiargyrou and O'Keefe, 2014; Liao et al., 2017). Studies have shown that miR-21 plays an important role in the above stages. Sun et al. discovered that in a rat osteoporotic fracture model, local injection of miR-21 nanocapsules promoted early bone repair in osteoporotic fractures, thereby accelerating bone healing into the molding phase earlier (Sun et al., 2020). Overexpression of miR-21 in a rat fracture model was found to accelerate endochondral ossification, increase the volume of callus and restore biomechanical strength of femur fractures (Sun et al., 2015). Liu et al. obtained the same results and found that miR-21 promotes fracture healing in rats by activating PI3K/AKT signaling pathway (Liu et al., 2019). However, in a rabbit tibial fracture model, miR-21 downregulation activated the ERK signaling pathway, which promoted the proliferation of osteoblasts and provided collagen and fibrous connective tissue required for fracture healing, thus shortening the formation time of bone callus and accelerating fracture healing (Sheng et al., 2019). The above different results show that miR-21 has a complex regulation mechanism in the process of fracture healing. Therefore, we need to analyze its role in fracture healing more deeply and comprehensively, to lay a good foundation for clinical research (Figure 2B; Table 3).

It is well known that osteoporosis increases the risk of fracture. Clinical studies have reported that miR-21 is also differentially expressed in patients with osteoporotic fracture and is related to bone turnover markers. This represents a possible association of miR-21 with an increased risk of osteoporotic fractures, while providing a new target for the prevention and treatment of osteoporotic fractures. The study of Seeliger et al. and Kelch et al. found that miR-21 was significantly up-regulated in patients with osteoporotic hip fracture (Seeliger et al., 2014; Kelch et al., 2017), and Panach et al. got the same results, in further, they showed that miR-21-5p was positively correlated with serum C-telopeptide (CTx) levels (Panach et al., 2015). Zarecki et al. detected significant upregulation of miR-21 in patients with vertebral fractures and low BMD, but they did not find any correlation between miR-21-5p and CTx (Zarecki et al., 2020). The findings of Yavropoulou et al. were contrary to the three studies mentioned above, and serum miR-21-5p levels were lower in patients who had suffered at least one vertebral fracture compared to those who had no fracture (Yavropoulou et al., 2017) (Table 4).

These opposite results may be related to the different control groups selected in the study population. Seeliger et al. and Kelch et al. chose a control group that included patients with fractures, while Panach et al. chose a control group of women with severe hip osteoarthritis requiring hip prosthesis implantation. Zarecki et al. and Yavropoulou et al. used postmenopausal women with normal BMD values and no fractures as a control group. Both fracture and OA affect miR-21 expression *in vivo*. In addition, Panach et al. found that there was a positive correlation between miR-21-5p and CTx levels, which also reflected that miR-21 was closely related to osteoclast production, but Zarecki et al. did not find any correlation between miR-21-5p and CTx, which indicated that the correlation between bone transition markers and miR-21 was not consistent, so more large-scale studies may be needed to further confirm the correlation between them.

## The Regulatory Role of miR-21 in OA

OA is a chronic debilitating disease that affects millions of people around the world (Ghouri and Conaghan, 2020). Joint pain, stiffness, swelling and dysfunction caused by it have brought great physical and mental damage to patients. Progressive destruction of articular cartilage is a major feature of OA (Sun et al., 2021).

MiR-21 is reported to be differentially expressed in OA cartilage and regulates cartilage degeneration. Clinical studies have shown that miR-21 is expressed at lower levels in cartilage from OA patients compared to normal cartilage biopsy, and that inhibition of miR-21 led to apoptosis of chondrocytes and degeneration of cartilage (Song et al., 2014). In contrast, Wang et al. identified significant upregulation of miR-21-5p expression in OA cartilage tissue compared to trauma patients without a history of OA (Wang et al., 2019). Zhang et al. reported that the expression level of miR-21 in the cartilage of patients with OA is higher than that of traumatic amputees. Meanwhile, they investigated the effect of miR-21 on chondrogenesis in a chondrocyte cell line and found that miR-21 promotes OA pathogenesis by targeting GDF-5. MiR-21 inhibits the expression of GDF-5 and its overexpression attenuates the progression of OA (Zhang et al., 2014). This finding was validated in the mouse temporomandibular joint osteoarthritis (TMJOA) model, where Zhang et al. claimed that knockdown of miR-21-5p reduced cartilage matrix degradation in TMJOA by targeting GDF-5 (Zhang A. et al., 2020). Also in the mouse TMJOA model, researchers discovered that miR-21-5p knockout mice had less temporomandibular joint cartilage destruction than wild-type mice, and *in vitro* experiments showed that miR-21-5p promotes extracellular matrix degradation and angiogenesis in TMJOA by suppressing the expression of target gene SPRY1, which in turn promotes the development of TMJOA (Ma et al., 2020). Furthermore, miR-21-5p upregulation also initiates and promotes OA by targeting FGF18, and intra-articular injection of antagomiR-21 attenuates cartilage degeneration in OA model mice, suggesting that targeting miR-21-5p is a promising option for the treatment of OA (Wang et al., 2019). However, the laboratory findings of Zhu et al. were not consistent with the above, as they discovered that miR-21-5p was significantly downregulated in OA chondrocytes and, more importantly, that miR-21-5p expression levels were negatively correlated with cartilage degeneration. Upregulation of miR-21-5p in OA chondrocytes improved changes in cartilage extracellular matrix-associated factors. These results suggest that miR-21-5p can act as a disease modifier in OA and play an important role in the pathological development of OA (Zhu et al., 2019).

Pain is the most prominent symptom in patients with OA (Abramoff and Caldera, 2020). MiR-21 was also associated with chronic pain in OA. MiR-21 was highly expressed in the synovial tissue and synovial fluid of OA model rats, and extracellular miR-21 released from synovial tissue caused knee joint pain in OA model rats through activation of TLR7. Notably, intra-articular injection of miR-21 inhibitors or TLR7-9 antagonists alone provided the long-term relief of pain in OA model rats. Therefore, extracellular miR-21 may be a possible target for OA pain treatment (Hoshikawa et al., 2020) (Figure 2B; Tables 3, 4).

## MiR-21 Involvement in the Diagnosis and Treatment of Osteonecrosis

Osteonecrosis is a common and refractory disease in orthopedics, which is caused by temporary or permanent interruption of blood supply in the affected bone area, resulting in bone structure collapse, joint pain and loss of related function (Lespasio et al., 2019).

Abnormal expression of miR-21 in osteonecrosis may be relevant to the diagnosis and treatment of osteonecrosis. MiR-21-3p (Another mature sequence, from the 3′ end of the miR-21 stem-loop precursor, is the miR-21 passenger strand) was discovered to be upregulated in BMMSCs in mice with steroid-induced osteonecrosis of the femoral head (SIONFH) (Wang et al., 2015). In the rat SIONFH model, miR-21-5p expression was also significantly upregulated, and it was further found circular RNA PVT1 attenuated SIONFH through regulation of the miR-21-5p-mediated Smad7/TGFβ signaling pathway (Hao et al., 2021). Yang et al. established rat bisphosphonate-related osteonecrosis of the jaw (BRONJ) model and found that miR-21 expression was upregulated in the serum of BRONJ rats, and miR-21 together with miR-23 and miR-145 could be used as a combined indicator for diagnosing or predicting the initiation and development of BRONJ. They obtained the same result in the serum of BRONJ patients (Yang et al., 2018). Another clinical study detected the total RNAs of circulating lymphocytes in healthy people and in multiple myeloma patients with BRONJ, they found that the expression profile of miRNA changed, and the expression level of 14 miRNAs increased in multiple myeloma patients with BRONJ. Targeting these miRNAs can provide a new opportunity for the prevention or treatment of BRONJ, and miR-21 is one of them (Musolino et al., 2018). In addition, miR-21 is also involved in osteocyte apoptosis in osteonecrosis. Kuang et al. showed that human Wharton’s jelly of umbilical cord mesenchymal stem cells (hWJ-MSCs) derived exosomes inhibit osteocyte apoptosis in glucocorticoid-induced osteonecrosis of the femoral head (GIONFH) in rats, and this effect was achieved through the miR-21-PTEN-AKT signaling pathway, which provides a new idea for the treatment of GIONFH (Kuang et al., 2019) (Figure 2B; Tables 3, 4).

## Conclusions and Perspectives

To sum up, miR-21 is a multi-target miRNA that plays an important role in bone metabolism, affecting the differentiation of osteoblasts and osteoclasts, and is closely related to osteopathic diseases such as osteoporosis, fracture, osteoarthritis and osteonecrosis. Firstly, there is differential expression of miR-21 in patients with osteoporosis, which can not only be used as a new biomarker for the diagnosis of osteoporosis, but also provide a new potential target for the treatment of osteoporosis. Secondly, miR-21 is also differentially expressed in patients with osteoporotic fractures, which may be related to the increased risk of osteoporotic fracture. MiR-21 can also promote fracture healing through a variety of mechanisms. Thirdly, in OA, miR-21 is differentially expressed in articular cartilage and thus regulates cartilage degeneration, providing a new target for the treatment of OA, and miR-21 is also associated with chronic pain caused by OA. Finally, miR-21 also showed changes in expression levels in osteonecrosis, which suggests that miR-21 may be relevant to the diagnosis and treatment of osteonecrosis. The function of miR-21 is complex and controversial, and the development, progression, and treatment of these orthopedic diseases is also a complex biological process that involves multiple cell types, multiple signaling pathways, and changes in the expression of related factors. Furthermore, clinical studies are inconsistent due to individual differences, many variables and difficulty to control, etc. Therefore, to further clarify the effect of miR-21 on osteoblasts and osteoclasts and its role in the above orthopedic diseases needs to be studied in depth.

## Glossary

ACVR2B: activin receptor type IIB
AGO: Argonaute
ALP: alkaline phosphatase
BMP9: Bone morphogenetic protein 9
BMMSCs: bone marrow mesenchymal stem cells
BMD: bone mineral density
BMSCs: bone marrow stromal cells
BRONJ: bisphosphonate-related osteonecrosis of the jaw
CTx: C-telopeptide
DGCR8: DiGeorge critical region 8
ERK: extracellular signal-regulated kinase
ERK-MAPK: extracellular signal–regulated kinase-mitogen-activated protein kinase
FasL: fas ligand
FGF18: fibroblast growth factor 18
GDF-5: growth differentiation factor 5
GIONFH: glucocorticoid-induced osteonecrosis of the femoral head
GSK3β: Glycogen synthase kinase 3β
HPDLSCs: human periodontal ligament stem cells
HUMSCs: human umbilical cord mesenchymal stem cells
M-CSF: macrophage colony-stimulating factor
miR-21: miRNA-21
MiRNAs: MicroRNAs
MMCs: murine multilineage cells
MSCs: mesenchymal stem cells
MT1-MMP: Membrane type 1 metalloprotease
OPG: osteoprotegerin
OPN: osteocalcin
OSX: Osterix
OVX: ovariectomy
PAOO: periodontal accelerate osteogenesis orthodontics
PDCD4: programmed cell death 4
PDLCs: periodontal ligament cells
PI3K: phosphatidylinositol 3-kinase
PLAP-1: periodontal ligament-associated protein-1
PMOP: postmenopausal hypoestrogenic women with osteoporosis
pri-miRNA: primary miRNA
Pre-miRNA: precursor miRNA
PTEN: phosphatase and tensin homologue deleted on chromosome 10
RANKL: receptor activator of nuclear factor κB ligand
RECK: reversion-inducing cysteine-rich protein with Kazal motifs
RISC: RNA-induced silencing complex
RUNX2: runt-related gene-2
SIONFH: steroid-induced osteonecrosis of the femoral head
SMAD7: small mothers against decapentaplegic 7
SMAD1/5/8: small mothers against decapentaplegic 1/5/8
SOX2: SRY sex determination SRY region Y-box2
SPRY1: Sprouty1
SPRY2: Sprouty2
SKP2: s-phase kinase associated protein 2
TLR7: Toll-like receptor
TM: tooth movement
TMJOA: temporomandibular joint osteoarthritis
TNF-α: tumor necrosis factor-α
